# Structural and Chemical Biology Approaches Reveal Isoform-Selective Mechanisms of Ligand Interactions in Mammalian Cryptochromes

**DOI:** 10.3389/fphys.2022.837280

**Published:** 2022-01-28

**Authors:** Simon Miller, Tsuyoshi Hirota

**Affiliations:** Institute of Transformative Bio-Molecules, Nagoya University, Nagoya, Japan

**Keywords:** cryptochromes, circadian clock, X-ray crystallography, small-molecule modulators, isoform selectivity

## Abstract

Cryptochromes (CRYs) are core components of the circadian feedback loop in mammals, which regulates circadian rhythmicity in a variety of physiological processes including sleep–wake cycles and metabolism. Dysfunction of CRY1 and CRY2 isoforms has been associated with a host of diseases, such as sleep phase disorder and metabolic diseases. Accumulating evidence for distinct roles of CRY1 and CRY2 has highlighted the need for CRY isoform-selective regulation; however, highly conserved sequences in CRY ligand-binding sites have hindered the design of isoform-selective compounds. Chemical biology approaches have been identifying small-molecule modulators of CRY proteins, which act in isoform-non-selective and also isoform-selective manners. In this review, we describe advances in our understanding of CRY isoform selectivity by comparing X-ray crystal structures of mammalian CRY isoforms in apo form and in complexes with compounds. We discuss how intrinsic conformational differences in identical residues of CRY1 and CRY2 contribute to unique interactions with different compound moieties for isoform selectivity.

## Introduction

The circadian clock is a biological timekeeper that regulates daily physiological rhythms in almost all organisms. In humans, sleep–wake behavior, hormone secretion, body temperature, and metabolism exhibit ~24-h circadian rhythms (Bass and Lazar, 2016). The suprachiasmatic nucleus (SCN) in the hypothalamus forms the central clock that orchestrates physiological rhythms in tissues and cells throughout the body *via* neuronal, hormonal, and behavioral signaling (Ralph et al., 1990; Reppert and Weaver, 2002). External stimuli, such as light-mediated activation of retinal ganglion cells, prompt the SCN to synchronize with environmental day–night cycles (Hatori and Panda, 2010). In contrast, feeding is the major timing cue for the peripheral clocks that locally regulate circadian rhythms in different tissues (Schibler et al., 2003).

At the core of the circadian clock, CLOCK and BMAL1 transcription factors form a heterodimer that binds to E-box elements and activates the transcription of thousands of genes regulating metabolism and other outputs (Rey et al., 2011; Koike et al., 2012). CLOCK-BMAL1 also activates *Period* (*Per1* and *Per2*) and *Cryptochrome* (*Cry1* and *Cry2*) genes, whose protein products repress CLOCK-BMAL1 to form a transcription–translation negative feedback loop (Takahashi, 2017). Alleviation of CLOCK-BMAL1 repression is regulated by proteasomal degradation of PER and CRY proteins, which is enhanced by post-translational modifications. Casein kinase I (CKI) phosphorylates PERs for degradation (Narasimamurthy and Virshup, 2021), and CKI inhibitors cause period lengthening of behavioral rhythms (Meng et al., 2010; Lee et al., 2019). In contrast, AMP-activated protein kinase (AMPK)- and glycogen synthase kinase-3β (GSK-3β)-dependent phosphorylation of CRYs regulate their degradation (Lamia et al., 2009; Kurabayashi et al., 2010). Proteasomal degradation of CRYs is mediated by ubiquitination *via* the E3 ligase FBXL3 (Busino et al., 2007; Godinho et al., 2007; Siepka et al., 2007), whereas FBXL21 induces CRY stabilization (Hirano et al., 2013; Yoo et al., 2013). The balance of CRY degradation and stabilization is also important for the regulation of circadian period length, such that degradation facilitates a new cycle to begin, and stabilization prolongs the current cycle. Both CRY1 and CRY2 isoforms can repress CLOCK-BMAL1 as part of a large complex with PER proteins, but only CRY1 is able to form a late repressive complex with CLOCK-BMAL1 in the absence of PERs (Koike et al., 2012; Michael et al., 2017; Rosensweig et al., 2018; Fribourgh et al., 2020). Furthermore, CRY2, but not CRY1, promotes c-MYC degradation *via* the recruitment of FBXL3 (Huber et al., 2016). This and other accumulating evidence have shown distinct regulatory roles of CRY1 and CRY2 isoforms.

CRY dysfunction has been implicated in diseases, such as circadian sleep disorders (Hirano et al., 2016; Patke et al., 2017) and diabetes (Zhang et al., 2010; Lamia et al., 2011). Furthermore, CRY1 and CRY2 isoforms have been differentially associated with cancer and may function as pro- or anti-tumorigenic factors depending on the type of cancer (Chan and Lamia, 2020; Chan et al., 2021; Shafi et al., 2021). CRYs therefore represent attractive drug targets for the treatment of CRY-mediated and circadian clock-related diseases. Because dysfunctional CRY1 and CRY2 appear to have overlapping and distinct associations with diseases, isoform-selective CRY-targeting compounds, in addition to isoform-non-selective compounds, would facilitate the understanding of molecular mechanisms underlying CRY functions to form the basis of therapeutics. In this review, we will introduce the development of small-molecule modulators of CRY proteins by chemical biology approaches, and how structural biology (Box 1) has enhanced our understanding of isoform-selective mechanisms that regulate compound interactions and activity.

BOX 1 **Macromolecular structure determination**

X-ray crystallography is a technique that enables the determination of tertiary protein structures at an atomic level. To determine CRY structures, purified CRY proteins are mixed with precipitant solutions and positioned (either sitting or hanging drops) over a reservoir containing a buffer, salt, and precipitant. Vapor diffusion removes water from the protein-containing drop increasing protein concentration and reducing solubility and thus driving it into a supersaturated state where crystal nucleation can occur. Protein crystals are cryoprotected in solutions containing high concentrations of a precipitant, such as glycerol or polyethylene glycols (PEGs), mounted in cryo-loops, and flash-cooled in liquid nitrogen to produce a vitreous, frozen state that is free of crystalline ice. The cryo-loops containing protein crystals are mounted on a goniometer at a synchrotron facility and rotated in 0.1–1° increments in a monochromatic beam of hard X-rays at a specific wavelength (typically ~0.9–1.0 Å). Coherently scattered X-rays produce diffraction patterns that are recorded on a detector. The intensities of reflections are computationally processed and Fourier transforms are used to calculate structure factors that contain information regarding the amplitudes and phases of reflections (NB. Phase information is missing in the diffraction data and needs to be calculated from a protein model, isomorphous replacement with a heavy atom, or anomalous scattering from selenium incorporation or native sulfur-containing residues). Data processing produces a 3-D electron density map into which the protein and bound ligands are modeled.

## Identification of CRY-Targeting Compounds

**Figure 1:**
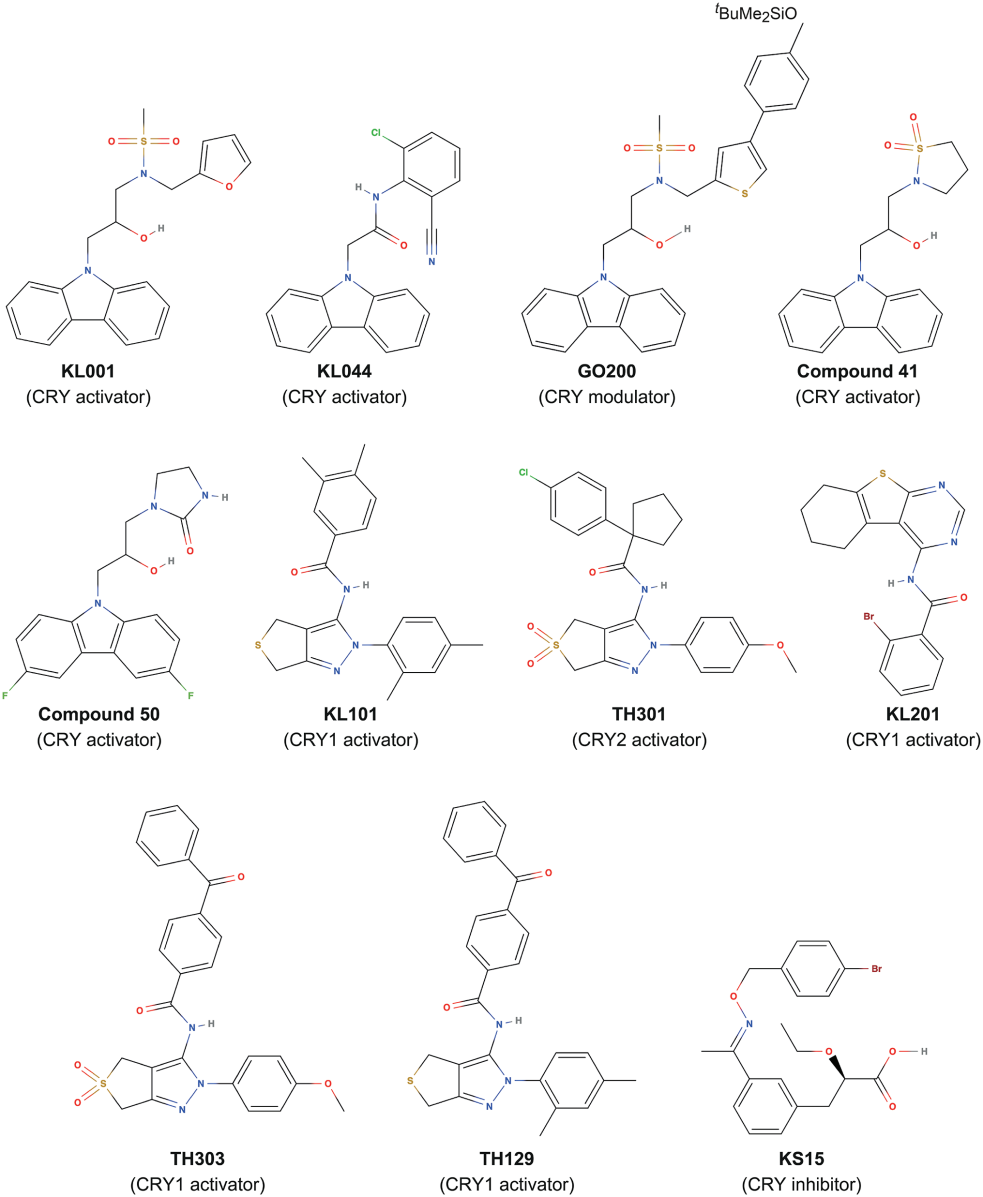
Small-molecule compounds that target CRY proteins.

The clock gene reporters *Bmal1-dLuc* and *Per2-dLuc* enable cellular circadian rhythms to be measured (Hirota and Kay, 2015). By evaluating the effects of compound libraries on the period, phase, and amplitude of circadian rhythms *via* phenotypic screening, modulators of clock function have been identified (Miller and Hirota, 2020). Among synthetic compounds with diverse structures, a carbazole derivative, KL001 (Figure 1), was found as a period-lengthening compound. Development of an affinity probe based on KL001, followed by affinity purification of interacting proteins and mass spectrometry analysis, revealed that KL001 was the first-in-class CRY modulator (Hirota et al., 2012). KL001 binds to the FAD pocket of CRY (Nangle et al., 2013; Miller et al., 2021) and competes with the C-terminal tail of FBXL3 (Xing et al., 2013), resulting in stabilization and activation of CRY for period lengthening (Hirota et al., 2012; Box 2). Development of KL001 derivatives resulted in the identification of KL044 as a more potent compound (Lee et al., 2015), and GO044 and GO200 as period-shortening compounds (Figure 1; Oshima et al., 2015). In addition to clock regulation, CRYs have been shown to inhibit glucagon and glucocorticoid-dependent induction of gluconeogenesis in the liver (Zhang et al., 2010; Lamia et al., 2011). Consistent with these findings, KL001 treatment of primary hepatocytes inhibits glucagon-dependent induction of gluconeogenesis (Hirota et al., 2012), and the orally available KL001 derivatives, compound 41 and compound 50 (Figure 1), have been shown to improve glucose tolerance and fasting blood glucose levels in diet-induced obese and diabetic mice (Humphries et al., 2016, 2018). CRY-targeting compounds therefore have therapeutic potential in the prevention of hyperglycemia. KL001 and its derivative are also efficacious in the treatment of glioblastoma, a malignant brain cancer, by reducing proliferation of patient-derived glioblastoma stem cells (Dong et al., 2019).

BOX 2 **Period regulation by CRY-targeting compounds**

Deletion of *Cry1* and *Cry2* genes results in period shortening and lengthening, respectively (van der Horst et al., 1999; Liu et al., 2007), suggesting opposite roles of these genes in period regulation. In contrast, CRY-activating compounds KL001, KL044, KL101, TH301, KL201, TH303, and TH129 cause period lengthening irrespective of their isoform selectivity (Hirota et al., 2012; Lee et al., 2015; Miller et al., 2020a,b; Kolarski et al., 2021a). Compound treatment in combination with mathematical modeling provided insights into the roles of CRY1 and CRY2 in period regulation (Hirota et al., 2012). FBXL3 is localized in the nucleus and degrades nuclear CRY1 and CRY2. Therefore, inhibition of FBXL3-dependent degradation by compounds results in nuclear accumulation of CRYs. Mathematical modeling predicted that stabilization of both nuclear CRY1 and CRY2 causes period lengthening. Consistent with this prediction, KL001 causes period lengthening in both *Cry1* and *Cry2* knockout cells (i.e., in the presence of only CRY2 or CRY1), and CRY1 or CRY2 isoform-selective compounds lengthen the period. Then, why do *Cry1* and *Cry2* knockouts result in opposite period change? Mathematical modeling suggested that the stronger repressor CRY1 and the weaker repressor CRY2 compete for a rate-limiting PER interaction in the cytosol to form a complex for nuclear localization. This competition determines the balance of CRY1 and CRY2 nuclear translocation, resulting in apparently opposite phenotypes of *Cry1* and *Cry2* knockouts: the weaker repressor CRY2 causes shorter period, and the stronger repressor CRY1 causes longer period. Compound treatment bypasses this rate-limiting step and directly stabilizes nuclear CRY1 and CRY2, resulting in period lengthening. Therefore, CRY1 and CRY2 have qualitatively similar roles in period regulation, while their potency is different. The molecular mechanisms of period shortening by GO200 (Oshima et al., 2015) and amplitude reduction by KS15 (Chun et al., 2014) are not clear and need further investigation.

Despite KL001 being an effective tool for CRY activation, it binds to CRY1 and CRY2 without preference, in other words, isoform non-selective, making it difficult to obtain insights into distinct functions of CRY isoforms. Very high sequence identity in the FAD pockets (see section “Structure of Mammalian CRYs”) in CRY1 and CRY2 has hindered the design and development of isoform-selective compounds. Analyses of period-lengthening compounds identified from phenotypic screens led to a breakthrough with the first discovery of CRY isoform-selective activators KL101 and TH301 (phenylpyrazole derivatives; Figure 1), which stabilize CRY1 and CRY2, respectively (Miller et al., 2020b; Box 2). Used as tools to activate CRY1 and CRY2, KL101 and TH301 helped to identify CRY-mediated activation of brown adipocyte differentiation (Miller et al., 2020b). Furthermore, a similar approach identified other CRY1 isoform-selective activators, the thienopyrimidine derivative KL201 (Miller et al., 2020a), and benzophenone derivatives TH303 and TH129 (Kolarski et al., 2021a), which together provide molecular tools to investigate CRY1 functions (Figure 1).

In addition to the activators, a CRY inhibitor was identified. The 2-ethoxypropanoic acid derivative KS15 (Figure 1) has been reported to target both CRY1 and CRY2 and block CRY-dependent inhibition of CLOCK-BMAL1 (Chun et al., 2014; Jang et al., 2018). KS15 has anti-proliferative effects on breast cancer MCF-7 cells by increasing *p53* and *Bax* gene expression (Chun et al., 2015). Considering that CRY1 and CRY2 have been associated with p53 suppression or degradation in other cancers (Chan et al., 2021; Jia et al., 2021) and that *Cry1* and *Cry2* knockout in *p53* mutant mice reduces cancer risk (Ozturk et al., 2009), KS15, or its derivatives (Jeong et al., 2021) may be efficacious in the treatment of several cancers, in addition to MCF-7 breast cancer. Together, these results identified CRYs as promising targets of small-molecule compounds.

## Structure of Mammalian CRYs

**Figure 2:**
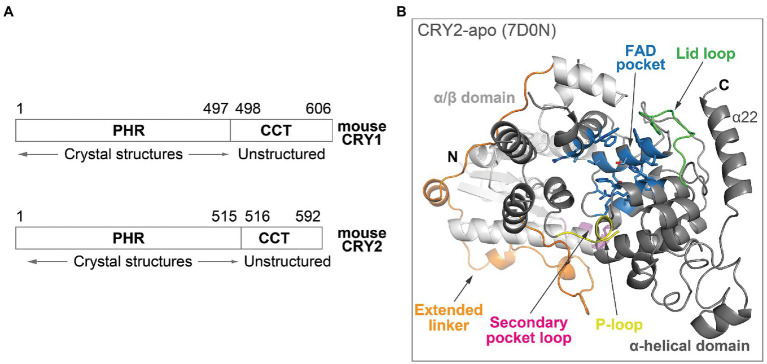
Overall structure of mouse CRY. **(A)** Domain organization of mouse CRY1 and CRY2. The PHRs are highly ordered with ~80% sequence identity between CRY1 and CRY2, whereas the CCTs have divergent sequences, variable lengths and are predicted to be structurally disordered. **(B)** The crystal structure of the PHR domain of CRY2-apo (PDB ID: 7D0N). The N-terminal α/β domain (white) and C-terminal α-helical domain (gray) are connected by an extended linker (orange). Important structural and regulatory features include: The FAD pocket (blue), lid loop (green), P-loop (yellow), and secondary pocket loop (magenta).

Cryptochromes belong to a photolyase/cryptochrome family of FAD-binding proteins, and the photolyase homology region (PHR) in mammalian CRYs (Figure 2A) is structurally related to photolyases. The PHR comprises several subdomains: An N-terminal α/β domain and a C-terminal α-helical domain, connected by an extended linker region (Figure 2B; Czarna et al., 2013; Xing et al., 2013; Miller et al., 2020b). Important structural and regulatory features in the PHR include: (1) The FAD pocket in mouse CRYs shares very high sequence identity between CRY isoforms. It has an open form that is accessible to molecules, rather than the closed form in photolyases that traps FAD in the pocket, and therefore can bind FAD, ligands, or the C-terminal tail of FBXL3 even after protein folding (Czarna et al., 2013; Xing et al., 2013; Miller et al., 2021); (2) the lid loop is juxtaposed to the FAD pocket and frequently interacts with residues in the FAD pocket in CRY crystal structures (Miller et al., 2021). Complex formation of CRY with PER proteins results in the restructuring of the loop and pocket residues (Nangle et al., 2014; Schmalen et al., 2014); (3) the P-loop can regulate circadian rhythms *via* phosphorylation (Ode et al., 2017), although the structural mechanisms are unknown due to flexibility and disorder of this loop; and (4) the secondary pocket binds to the PAS-B domain of CLOCK (Michael et al., 2017; Rosensweig et al., 2018). Several variant residues between CRY1 and CRY2 are located in the vicinity of the secondary pocket loop, resulting in a non-structured loop in CRY1, which facilitates the tighter binding of CRY1 to CLOCK PAS-B (Fribourgh et al., 2020). This enables the formation of a late repressive complex with CLOCK in the absence of PER (Koike et al., 2012).

In addition to the PHR domain, CRYs also contain a CRY C-terminal tail (CCT), which displays considerable sequence divergence between CRY1 and CRY2. A study utilizing cell-based CRY genetic rescue assays showed that the CCT is dispensable for rhythm generation, but regulates period length and rhythm amplitude (Khan et al., 2012). CCTs are mostly unstructured in secondary structure predictions, making their structural characterization challenging (Figure 2A; Parico and Partch, 2020). In contrast, the CCT in *Drosophila* CRY (dCRY) is much shorter than in mammalian CRYs, and its crystal structure has been determined (Czarna et al., 2013; Levy et al., 2013). dCRY CCT interacts with the lid loop and also directly with two key FAD pocket residues: H378 (equivalent to mouse CRY1 H355 and CRY2 H373) and W422 (equivalent to CRY1 W399 and CRY2 W417). H378 forms a bridge between the CCT and the FAD molecule, and both the protonation state and conformation of this His residue regulate CCT docking and undocking (Ganguly et al., 2016; Chandrasekaran et al., 2021). However, no mammalian apo form structures containing the CCT are available, so structural mechanisms of interactions among the FAD pocket, lid loop, and CCT are currently unknown. The region encoded by exon 11 of CRY1 in the CCT interacts with the PHR and regulates its affinity for CLOCK-BMAL1 (Parico et al., 2020). The CCT appears to be highly dynamic in nature and may regulate CRY activity by binding to different regions of the PHR to control circadian timing.

## Molecular Interactions of CRYs With Compounds

**Figure 3:**
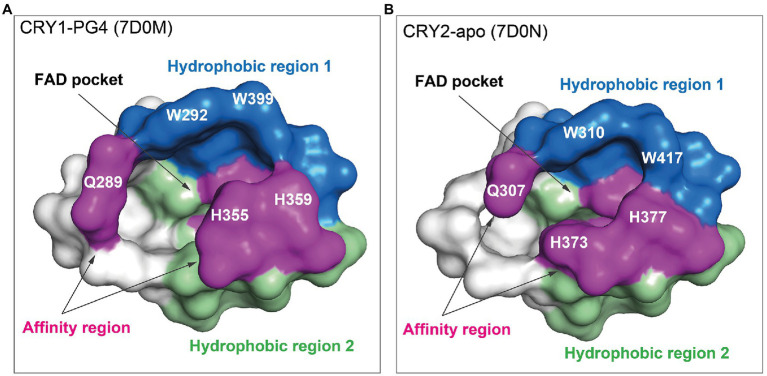
FAD pocket surfaces in CRY1 and CRY2. The FAD pocket can be divided into three subregions: Hydrophobic region 1 (blue), the affinity region (magenta), and hydrophobic region 2 (green). Pockets are shown for CRY1-PG4 [the PG4 molecule (not shown) was present in the crystallization buffer and bound non-specifically in the FAD pocket; PDB ID: 7D0M] **(A)** and CRY2-apo (7D0N) **(B)**.

**Figure 4:**
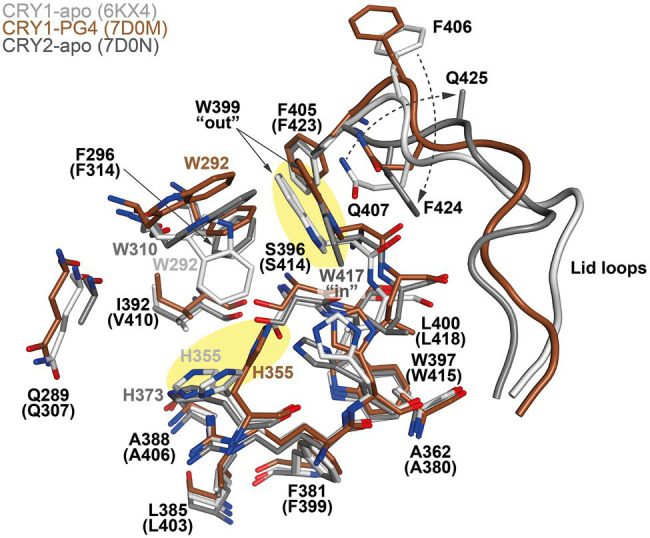
Conserved residues in the FAD pockets and lid loops of CRY1 and CRY2 intrinsically adopt distinct conformations. Superposition of CRY1-apo (PDB ID: 6KX4; white), CRY1-PG4 (7D0M; brown; PG4 molecule not shown) and CRY2-apo (7D0N, gray) shows heterogeneity in the conformations of the FAD pocket residues CRY1 H355 and W399 (yellow shading), and the lid loop residues F406 and Q407 (CRY2 H373, W417, F424, and Q425, respectively). The opposite conformations of the CRY1 W399 and CRY2 W417 gatekeepers form differential interactions with reversed lid loop conformations (indicated by dotted arrows). CRY1 W292 displays some flexibility in CRY1 structures and occupied a “down” conformation in CRY1-apo (6KX4), but is repositioned to accommodate compounds (Figures 5–7).

The design of isoform-selective compounds has been hindered by almost identical sequences in the FAD pockets of CRY1 and CRY2 and their highly similar overall PHR structures. Recent crystal structures of mouse CRY1-PG4 [PDB: 7D0M; apo-like structure with a weakly bound cryoprotectant tetraethylene glycol (PG4) in the FAD pocket] and CRY2-apo (PDB: 7D0N), however, have provided insights into intrinsic structural differences in their FAD pockets and lid loops (Miller et al., 2021). The FAD pocket is composed of three subregions: (1) Hydrophobic region 1 consisting of mouse CRY1 residues W292, F296, W399, and L400 (W310, F314, W417, and L418 in CRY2); (2) Affinity region: CRY1 residues Q289, H355, H359, and S396 (Q307, H373, H377, and S414 in CRY2); and (3) Hydrophobic region 2: CRY1 residues R358, A362, F381, L385, A388, I392, and W397 (R376, A380, F399, L403, A406, V410, and W415 in CRY2; Figures 3, 4). These 15 residues represent residues that most often interact with compounds, and CRY1 S396/CRY2 S414 in the affinity region form a canonical hydrogen bond in all compound structures. Superposition of CRY1-apo (PDB: 6KX4), CRY1-PG4, and CRY2-apo shows how fully conserved residues adopt different conformations (Miller et al., 2021; Figure 4). Most notable are CRY1 W399 “out” and corresponding CRY2 W417 “in” conformations (these Trp residues are referred to as the gatekeeper due to interactions or steric clashes with compound functional groups depending on their “out” or “in” orientations; see below); CRY1 H355 corresponding to CRY2 H373; and lid loop residues CRY1 F406-Q407 and CRY2 F424-Q425.

X-ray crystal structures represent the gold standard for elucidating molecular conformations and interactions, but their structures are predominantly static and can have conformational restraints imposed by crystal packing (interactions between protein molecules that form the crystal lattice). It is therefore important to evaluate the integrity of protein structures with supporting methods and analyses. Molecular dynamics (MD) simulations computationally analyze energetically favorable conformations in dynamic structures. Comparisons of X-ray crystal structures and MD simulations have shown agreement in the differential conformations of the gatekeepers and lid loops in CRY1 and CRY2, supporting that intrinsic conformational differences occur despite the high sequence similarity (Miller et al., 2021). In contrast, CRY1 H355/CRY2 H373 is more dynamic in CRY apo structures and did not show isoform-dependent differences. In the intrinsic “out” conformation in CRY1, the gatekeeper W399 interacts with Q407 in the lid loop, which helps to rigidify the N-terminal portion of the lid loop in combination with the canonical insertion of F405 into the auxiliary pocket located just behind hydrophobic region 1. In CRY2, F423 inserts into the auxiliary pocket in an equivalent manner to CRY1 F405, but the gatekeeper W417 adopts an “in” conformation and interacts with F424 in the lid loop. This stabilizes the N-terminal region of the loop in a different conformation to CRY1 and results in the lid loop residues CRY1 F406-Q407 and CRY2 F424-Q425 rotating ~180° relative to each other (Miller et al., 2021; Figure 4).

**Figure 5:**
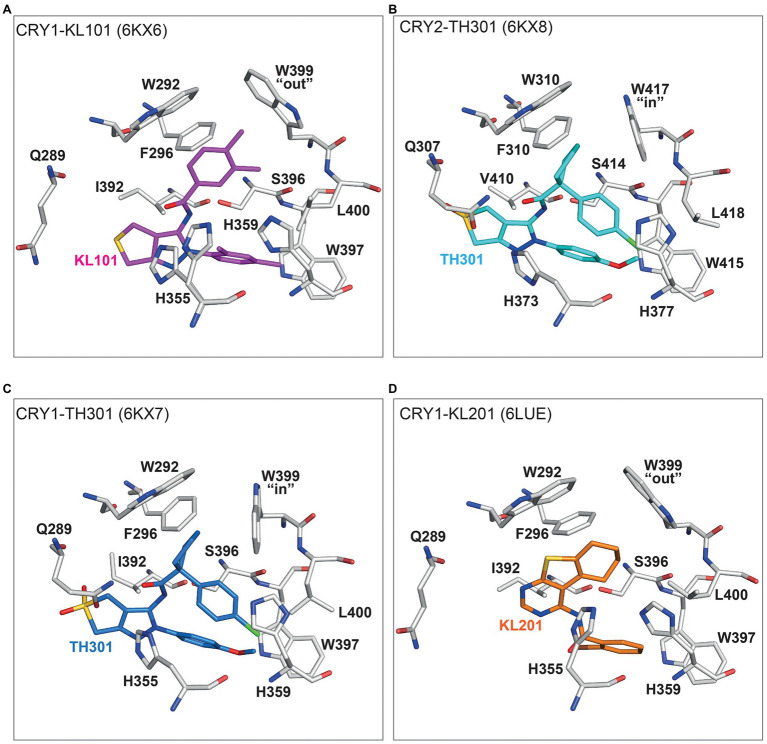
Binding modes of isoform-selective compounds in CRY1 and CRY2. **(A)** Crystal structure of CRY1-KL101 (PDB ID: 6KX6). Key interacting residues (white) and the KL101 compound (magenta) are shown. The upper dimethylphenyl located in hydrophobic region 1 and fitted to an intrinsically “out” gatekeeper W399 conformation. The lower dimethylphenyl located in hydrophobic region 2 and formed hydrophobic interactions with W397. H355 was not restrained by KL101 binding and transiently adopted the same conformation observed in CRY1-PG4 (Figure 4) and CRY1-KL201 **(D)**. **(B)** Crystal structure of CRY2-TH301 (6KX8). The cyclopentyl group of TH301 (cyan) binds in hydrophobic region 1 and forms a stacking interaction with the intrinsically “in” conformation of gatekeeper W417 (white). The methoxyphenyl group (in hydrophobic region 2) forms a hydrogen bond with W415. H373 adopted the same conformation as in CRY2-apo (Figure 4). **(C)** The binding of TH301 (blue) to CRY1 (white) induces a conformational change in CRY1-TH301 (6KX7). The small steric bulk of the cyclopentyl of TH301 induced a stacking interaction with the gatekeeper W399, which rotated from an intrinsic “out” conformation to an “in” conformation. The conformational freedom of H355 is restricted by the methoxyphenyl group, preventing H355 adopting a conformation observed in CRY1-KL101 **(A)** and CRY1-PG4 (Figure 4). **(D)** A unique binding mode of KL201 (orange) to CRY1 (white) in CRY1-KL201 (6LUE). The fused tricyclic thienopyrimidine and cyclohexyl moiety of KL201 binds to hydrophobic region 1 of the FAD pocket. In this configuration, the compound fits to an intrinsically “out” gatekeeper W399 conformation. H355 adopted a stable conformation similar to CRY1-PG4 (Figure 4) and CRY1-KL101 **(A)**, but distinct from CRY1-TH301 **(C)**. The bromophenyl group (essential for compound effect) located in hydrophobic region 2.

The intrinsic difference of the gatekeeper orientation explains well the isoform selectivity of KL101 and TH301. The phenylpyrazole groups of KL101 and TH301 occupied hydrophobic region 2 of the FAD pocket, although their binding positions were slightly offset due to substituent functional groups: a dimethylphenyl and a methoxyphenyl in KL101 and TH301, respectively, which formed distinct interactions with residue W397 in CRY1 and corresponding W415 in CRY2 (Figures 5A,B; Miller et al., 2020b). KL101 contains an upper dimethylphenyl that inserted into hydrophobic region 1 of CRY1 (PDB: 6KX6), and the steric bulk of the dimethylphenyl induced a slight outward push of the gatekeeper W399. Importantly, W399 retained the intrinsic “out” conformation observed in CRY1-PG4 and CRY1-apo structures (Figures 4, 5A). H355 also adopted a similar conformation to CRY1-apo and CRY1-PG4. In contrast, a notable conformational change occurred upon the binding of TH301 to CRY1 (PDB: 6KX7; Miller et al., 2020b). The gatekeeper rotated to an “in” position to form a stacking interaction with the cyclopentyl group (Figure 5C). The binding mode of TH301 in CRY2 (PDB: 6KX8) was almost identical to that in the CRY1-TH301 complex, but critically the CRY2 gatekeeper W417 required no conformational change, compared to CRY2-apo, to form a favorable stacking interaction with the cyclopentyl (Figure 5B). Furthermore, H355 in CRY1-TH301 adopted a different conformation to CRY1-PG4 and CRY1-KL101, whereas corresponding H373 in CRY2-TH301 retained the same conformation as in CRY2-apo (Figures 4, 5A,B). Overall, the results showed that KL101 and TH301 can bind to the intrinsic conformations of the FAD pockets in CRY1 and CRY2, respectively, accounting for their isoform-selective properties. In contrast, TH301 (selective to CRY2) induced large conformational changes in the FAD pocket of CRY1, indicative of less favorable interactions. Structure-guided point mutations in the lid loop residues CRY1 Q407 and CRY2 F424, which differentially interact with the CRY1 W399 and CRY2 W417 gatekeepers, resulted in inversed responses to KL101 and TH301 (Miller et al., 2021), supporting that differential intrinsic gatekeeper-lid loop conformations in CRY isoforms contribute to compound selectivity.

Structure activity relationships (SARs) of KL101 and TH301 derivatives provided insights into the functional groups required for optimal compound effects (Miller et al., 2020b). The *meta*-methyl group (interacted with the gatekeeper in hydrophobic region 1) and the *para*-methyl group of KL101 (interacted with hydrophobic residues in hydrophobic region 2) were important for compound effect, and their removal or substitution resulted in severely reduced activity. In TH301, the removal and substitution of the methoxy group (interacted with W397 in hydrophobic region 2) caused inactivity and reduced activity, respectively, and both the cyclopentyl and chlorophenyl groups were essential for activity.

The structure of CRY1 in complex with KL201 (PDB: 6LUE) revealed a unique interaction with a thienopyrimidine scaffold (Miller et al., 2020a). Compared to KL001 and KL044 (see below), KL201 appears to bind upside down in the FAD pocket with the fused tricyclic thienopyrimidine and cyclohexyl moiety occupying hydrophobic region 1, instead of hydrophobic region 2 (Figure 5D). In this orientation, KL201 fits to a gatekeeper “out” conformation. The exact mechanisms of CRY1 selectivity are not clear, but the thienopyrimidine scaffold has greater steric bulk than the functional groups of KL101 and TH301 that occupy hydrophobic region 1, and might cause a steric clash with the CRY2 gatekeeper “in” conformation or W310 (equivalent to CRY1 W292), which is closer to the center of the FAD pocket in CRY2. Another mechanism potentially regulating selectivity is the conformation of H355 in CRY1-KL201, which is very similar to CRY1-PG4 and CRY1-KL101, but different to CRY2-apo (Figures 4, 5D). SAR analyses of KL201 derivatives identified an essential role of the bromophenyl group (interacted with residues in hydrophobic region 2) in compound activity and a size-dependent effect of the cyclohexyl (interacted with W292 and W399; Miller et al., 2020a). Together, the crystal structures of CRY–compound complexes identified a mechanism whereby compounds compatible with intrinsic FAD pocket conformations of CRY1 and CRY2 show preferential binding. However, it is important to note that these compounds are only marginally selective in the context of PHR-only CRY constructs and require the CCT for full selectivity (Miller et al., 2020b, 2021; see below).

**Figure 6:**
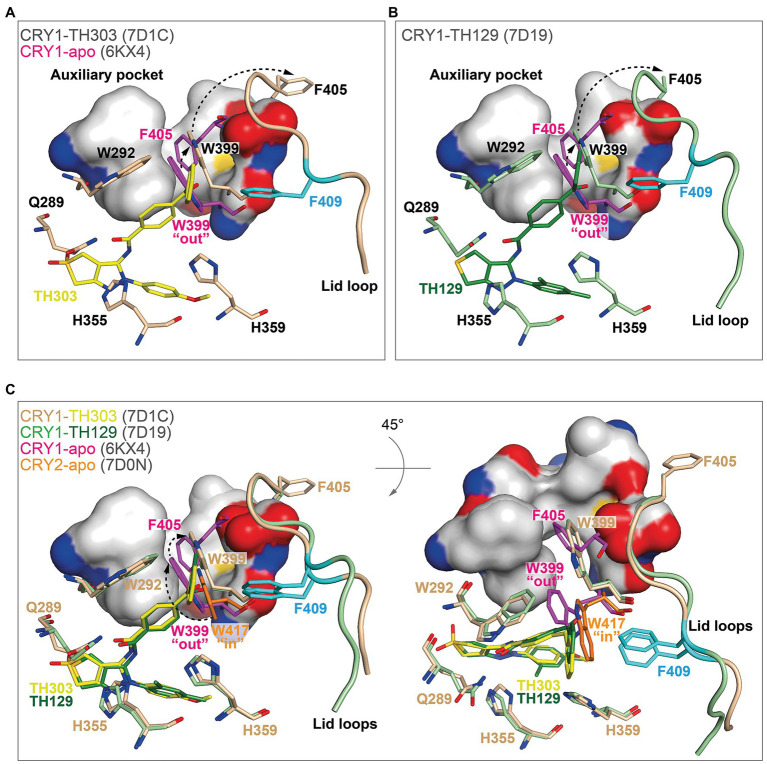
The binding of benzophenone derivatives to CRY1 induced gatekeeper and lid loop reordering. **(A,B)** The benzophenone moieties of TH303 (**A**, yellow, 7D1C) and TH129 (**B**, green, 7D19) form a severe steric clash with the intrinsic gatekeeper W399 “out” conformation of CRY1-apo (6KX4; magenta). Repositioning of W399 (beige and light green) into the auxiliary pocket (small arrow) induced steric overlap with F405 (intrinsically inserts into the auxiliary pocket), which relocated to a distal position (large arrow) causing rearrangement of the lid loop. The lid loop residue F409 (cyan) was repositioned to form a stacking interaction with the benzophenone. The surface is shown for the auxiliary pocket residues F295, F296, A299, F306, I314, M398, and S404. **(C)** Conformational isomerism of CRY1 and CRY2 gatekeepers may regulate TH303 and TH129 selectivity. Superposition of CRY2-apo (7D0N) gatekeeper W417 onto CRY1 crystal structures. The intrinsic CRY2 gatekeeper W417 “in” conformation would likely require two conformational changes to enter the auxiliary pocket (dotted arrows, left panel). A rotated view (45° around the X-axis) shows the large conformational change required for the gatekeeper to enter the auxiliary pocket (right panel), making it less energetically favorable in CRY2.

In contrast, TH303 and TH129 are selective to CRY1 PHR constructs missing the CCT (Kolarski et al., 2021a). TH303 and TH129 contained the same phenylpyrazole scaffold as KL101, but attached to a benzophenone substituent that inserted into hydrophobic region 1 in CRY1-TH303 (PDB: 7D1C) and CRY1-TH129 (PDB: 7D19) structures. In order to avoid a clash with the large steric bulk of the benzophenone moiety, the gatekeeper W399 underwent a considerable and unique conformational change where it inserted into the auxiliary pocket causing the ejection of F405 (Figures 6A,B). The removal of F405 from this pocket induced rearrangement of the lid loop and facilitated an interaction of F409 with the benzophenone. The benzoyl group in the benzophenone moiety of TH129 was critical for activity, and its substitution resulted in dramatically reduced period-lengthening activity. Furthermore, TH303 and TH129, but not KL101, showed reduced effects on a CRY1 F409A lid loop mutant (Kolarski et al., 2021a), supporting an interaction of the lid loop with the benzophenone. Superposition of TH303 and TH129 with CRY2-apo shows how a more severe steric clash would occur between the benzophenone and the gatekeeper W417 (Figure 6C). Moreover, the intrinsic “in” conformation of the gatekeeper in CRY2 would require a much larger conformational change to insert into the auxiliary pocket, which is likely to be energetically unfavorable. Taken together, TH303 and TH129 may impart isoform selectivity *via* large conformational changes to the gatekeeper and lid loop in CRY1.

**Figure 7:**
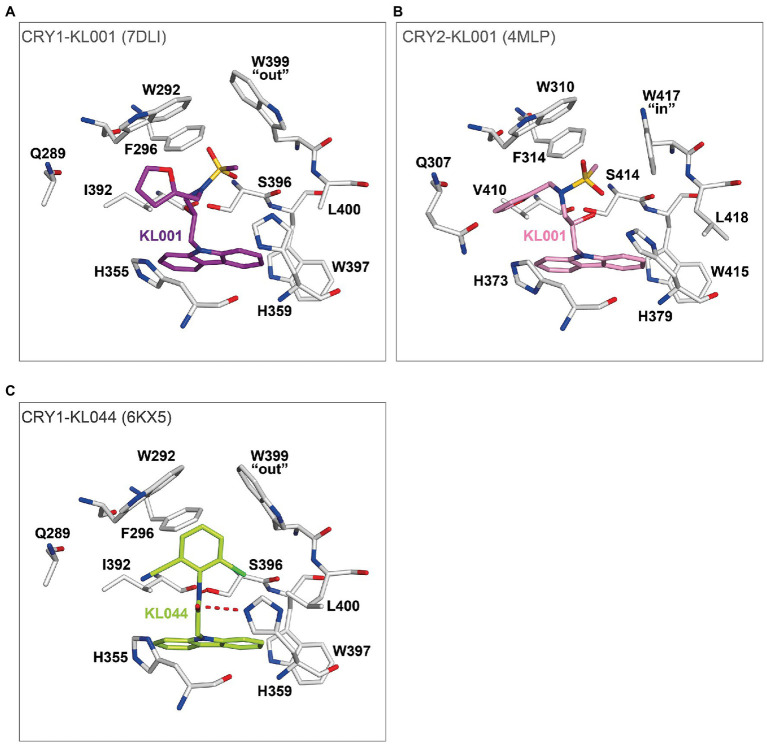
The binding modes of isoform non-selective CRY compounds. **(A)** Crystal structure of CRY1-KL001 (7DLI). The carbazole and hydroxypropyl linker of KL001 (purple) stably interacted with hydrophobic region 2 and the affinity region of CRY1 (white), respectively. In contrast, the methanesulfonamide and furan groups have greater flexibility and located in hydrophobic region 1 with an intrinsic gatekeeper W399 “out” conformation. **(B)** Crystal structure of CRY2-KL001 (4MLP). KL001 (pink) can bind to intrinsic FAD pocket conformations, including gatekeeper W417 “in” of CRY2 (white). **(C)** The potent CRY activator KL044 formed stable hydrogen bonds with the FAD pocket. The amide linker in KL044 (lime green) formed a hydrogen bond with H359 in addition to a canonical hydrogen bond with S396. The chlorobenzonitrile group formed hydrophobic interactions with W292, F296, and W399, and a CH–Cl interaction with L400 in hydrophobic region 1. Furthermore, the intrinsic gatekeeper W399 “out” conformation could accommodate the chlorobenzonitrile. The carbazole interacted with multiple residues in hydrophobic region 2. Together, KL044 had an optimized binding mode.

Crystal structures of the non-isoform-selective compound KL001 in complexes with CRY1 (PDB: 7DLI; Miller et al., 2021) and CRY2 (PDB: 4MLP; Nangle et al., 2013) revealed how this compound can bind to the FAD pocket of both CRY1 and CRY2 with minimal changes to intrinsic FAD pocket residues. The carbazole moiety and hydroxypropyl linker interacted with hydrophobic region 2 and the affinity region, respectively, and formed very similar binding modes in CRY1 and CRY2. In contrast, the more flexible methanesulfonamide and furan groups showed different binding modes in hydrophobic region 1 (Figures 7A,B). Importantly, the distinct “out” and “in” gatekeeper conformations of CRY1 and CRY2, respectively, can accommodate KL001, suggesting a mechanism for its isoform non-selective properties. A more potent derivative KL044 (Lee et al., 2015) formed strong interactions with FAD pocket residues in complex with CRY1 (Miller et al., 2020b; Figure 7C). Similar to KL001, the carbazole group in KL044 stably interacted with hydrophobic region 2. A chlorobenzonitrile moiety inserted into a hydrophobic annulus in hydrophobic region 1 interacting with W292, F296, and the gatekeeper W399 and also formed a CH–Cl interaction with L400. An amide linker connecting the bottom carbazole and top chlorobenzonitrile moieties formed a hydrogen bond with H359, in addition to a canonical interaction with S396. These interactions resulted in an optimized binding orientation of KL044 in the FAD pocket, accounting for its higher potency. The binding mode of KL044 in CRY2 has not been determined, but the small steric bulk of the chlorobenzonitrile may be able to avoid a steric clash with the intrinsic “in” conformation of the gatekeeper W417.

Overall, crystal structures of CRYs in complexes with small-molecule activators have provided structural insights into their mechanisms of action. In contrast, there is currently no structural information available for the mechanisms by which CRY inhibitors, for example, KS15, exhibit their effects. Such structures could facilitate the development of new inhibitor derivatives, providing new tools for CRY regulation.

## Role of CCTs in Isoform Selectivity

Although structural information of the CCT is not available because of its disordered nature, functional assays have provided insights into the role of the CCT, in combination with the lid loop and gatekeeper, in compound selectivity. Chimeric CRY1 and CRY2 proteins containing their respective PHRs but with segment-swapped CCTs from their opposite isoforms are non-responsive to KL101 and TH301 (Miller et al., 2020b), demonstrating that CCT interactions with their respective PHRs differ between CRY isoforms. In contrast, CCT-swapping in combination with lid loop mutations (CRY1 Q407A and CRY2 F424A) can influence compound selectivity (Miller et al., 2021), suggesting their interplay. Segment swapping and truncations of CRY CCTs have determined that the region encoded by exon 10 is required for the effects of KL101 and TH301 (Miller et al., 2020b). Interestingly, unlike in mammalian cells, KL101 and TH301 did not change the circadian period in zebrafish, possibly due to markedly different sequences in the exon 10 region of zebrafish CRY1 and CRY2, compared to mammalian CRYs (Iida et al., 2021). This observation indicates a species-dependent difference in the effects of KL101 and TH301 and supports an important role of the exon 10 region in their effects. Furthermore, a hydrophobic patch (Phe-Met-Gly-Tyr) in human CRY1 exon 10 is reported to interact with the PHR by nuclear magnetic resonance spectroscopy (Parico et al., 2020). Together, these findings suggest a mechanism where the CCT might impart compound selectivity by interacting with residues in the FAD pocket and lid loop, and/or directly with compounds. It is possible that divergent CCTs in CRY1 and CRY2 bind to and stabilize intrinsically different FAD pocket and lid loop conformations observed in CRY PHR structures or that CCT binding induces conformational rearrangement of key pocket residues and the lid loop. However, specific mechanisms remain elusive, and crystal structures of full-length CRY proteins with and without bound compounds will be pivotal in the future elucidation of these mechanisms.

## Concluding Remarks

Intrinsic differences in CRY1 and CRY2 structures have shed light on the isoform-selective binding of compounds. The differential conformations of FAD pocket residues are analogous to a lock, and compounds that show compatible binding to these conformations are analogous to a key. Further interactions among the FAD pocket, lid loop, and CCT appear to stabilize distinct conformations and impart isoform selectivity. Compounds with opposite selectivity that require conformational changes to intrinsic pocket residues, most likely incur steric hindrance to pocket entry or less energetically favorable interactions. Despite these advances in our understanding of CRY regulation by small-molecule compounds, there are many mechanisms yet to be structurally determined. In particular, molecular interactions between the PHR and CCT governing isoform selectivity, and intrinsic differences in highly conserved PHR residues that regulate the binding of PHR isoform-selective compounds, such as TH303 and TH129, still require elucidation. Furthermore, the regulatory mechanisms of period-shortening compounds, such as GO200, are poorly understood. Future research should determine these critical regulatory mechanisms. The understanding of compound-binding modes will facilitate the derivatization and development of new compounds with optimal binding properties. The development of improved isoform-specific CRY modulators could provide new insights into distinct CRY1 and CRY2 functions, as well as more efficacious treatment of clock-related diseases due to the activation or inhibition of only one CRY isoform. In addition, derivatization of CRY1-selective TH129 by substituting the benzophenone with an azobenzene moiety has enabled reversible regulation of CRY1 function with light (Kolarski et al., 2021a). This shows promise in the field of circadian photopharmacology, where visible light may be used to regulate circadian rhythms by targeting only specific tissues and cells (Kolarski et al., 2019, 2021b). In summary, chemical and structural biology have furthered our understanding of how evolutionarily conserved CRY isoforms can be selectively targeted by small-molecule compounds and provided a rationale for new compound development to optimize interactions with the conformational isomerism present in CRY1 and CRY2 structures.

## Author Contributions

SM and TH wrote this review. All authors contributed to the article and approved the submitted version.

## Funding

TH received support from JSPS Grants 20K21269 and 21H04766; Takeda Science Foundation; Uehara Memorial Foundation; Tokyo Biochemical Research Foundation; and Hitachi Global Foundation.

## Conflict of Interest

The authors declare that the research was conducted in the absence of any commercial or financial relationships that could be construed as a potential conflict of interest.

## Publisher’s Note

All claims expressed in this article are solely those of the authors and do not necessarily represent those of their affiliated organizations, or those of the publisher, the editors and the reviewers. Any product that may be evaluated in this article, or claim that may be made by its manufacturer, is not guaranteed or endorsed by the publisher.
